# Modeling Multidimensional Public Opinion Polarization Process under the Context of Derived Topics

**DOI:** 10.3390/ijerph18020472

**Published:** 2021-01-08

**Authors:** Tinggui Chen, Yulong Wang, Jianjun Yang, Guodong Cong

**Affiliations:** 1 School of Statistics and Mathematics, Zhejiang Gongshang University, Hangzhou 310018, China; 2 School of Management and E-Business, Zhejiang Gongshang University, Hangzhou 310018, China; yulong1105@yeah.net; 3 Department of Computer Science and Information Systems, University of North Georgia, Oakwood, GA 30566, USA; Jianjun.Yang@ung.edu; 4 School of Tourism and Urban-Rural Planning, Zhejiang Gongshang University, Hangzhou 310018, China; cgd@mail.zjgsu.edu.cn

**Keywords:** multi-dimensional public opinion polarization, topic derivation, external intervention information, topic correlation coefficient

## Abstract

With the development of Internet technology, the speed of information dissemination and accelerated updates result in frequent discussion of topics and expressions of public opinion. In general, multi-dimensional discussion topics related to the same event are often generated in the network, and the phenomenon of multi-dimensional public opinion polarization is formed under the mutual influence of groups. This paper targets the phenomenon of multi-dimensional public opinion polarization under topic-derived situations as the research object. Firstly, this paper identifies the factors influencing multi-dimensional public opinion polarization, including the mutual influence of different topic dimensions and the interaction of viewpoints within the same topic. Secondly, the topic correlation coefficient is introduced to describe the correlation among topics in different dimensions, and the individual topic support degree is used to measure the influence of topics in different dimensions and that of information from external intervention on individual attitudes. Thirdly, a multi-dimensional public opinion polarization model is constructed by further integrating multi-dimensional attitude interaction rules. Finally, the influence of individual participation, topic status, topic correlation coefficient and external intervention information on the multi-dimensional public opinion polarization process is analyzed through simulation experiments. The simulation results show that: (1) when there is a negative correlation between multi-dimensional topics, as the number of participants on different dimensional topics becomes more consistent, the conflict between multi-dimensional topics will weaken the polarization effect of overall public opinion. However, the effect of public opinion polarization will be enhanced alongwith the enhancement in the confidence of individual opinions. (2) The intervention of external intervention information in different dimensions at different times will further form a multi-dimensional and multi-stage public opinion polarization, and when the multi-dimensional topics are negatively correlated, the intervention of external intervention information will have a stronger impact on the multi-dimensional and multi-stage public opinion polarization process. Finally, the rationality and validity of the proposed model are verified by a real case.

## 1. Introduction

After the outbreak of an event, relevant information will spread rapidly on the Internet. Affected by information with multiple tuples, netizens’ comments are multidimensional, meaning the comments are made from various perspectives or vary over time, thus leading to multidimensional topics. The interaction of viewpoints about multidimensional topics makes public opinion escalate to a climax, resulting in multidimensional public opinion polarization. In real life, most hot events show multi-dimensional characteristics, such as the “Liu Qiangdong sexual assault incident”. Netizens’ views on the event show multiple dimensions. At the beginning, most netizens criticized Liu Qiangdong’s sexual assault. Since then, with more disclosure of information, netizens have talked about its derivative subject:“Liu had been framed”, “Sino-US trade war trap”. With the development of events, the multidimensional public opinion polarization phenomenon is formed, and thus led to a pattern of network violence, impacting on social harmony and stability. Based on this, it is of great theoretical and practical significance to study the multi-dimensional public opinion polarization process in the topic derivation context.

There is limited study on the multi-dimensional public opinion polarization phenomenon in a topic-derived context, while most studies conduct research from a single dimension of network public opinion topics and use macro statistics [1,2] or a mathematical modeling method [3,4] to analyze the formation process. In fact, after the outbreak of an event, affected by information with multiple tuples, discussion topics with multiple dimensions tend to be derived [5]. However, netizens’ debates on topics with different dimensions will enable the polarization of online public opinions to be multi-dimensional. Based on this, from the perspective of topic derivation, the paper studies the interaction mechanism between multi-dimensional topics and analyzes the internal and external factors that form multi-dimensional public opinion polarization, so as to have a more comprehensive understanding of its formation mechanism. Hot topics are not limited to a single dimension, but affected by multi-dimensional information. The evolution of multidimensional attitudes is not only affected by the interaction of internal views on the same dimensional topic but is also affected by other dimensional topics and external intervention information. Under joint action, individuals will constantly adjust their own attitudes, thus forming the phenomenon of multi-dimensional public opinion polarization. Based on this, this paper starts from the topic derivation context, expands the hot topic category from a single dimension to multiple dimensions, introduces the topic correlation coefficient and individual topic support index, and further integrates the multi-dimensional attitude interaction rules to build a multi-dimensional public opinion polarization model in the topic derivation context. Finally, the influence of the individual participation topic status, topic correlation coefficient, individual viewpoint confidence and external intervention information on the multi-dimensional public opinion polarization process is discussed through simulation experiments, and the rationality and effectiveness of the model proposed in this paper are verified by a real case.

The structure of this paper is as follows: Section 2 is a literature review. Section 3 constructs the multi-dimensional public opinion polarization model under the topic derived situation. Section 4 analyzes the influence of some main parameters on the multi-dimensional public opinion polarization process through a simulation experiment. Section 5 verifies the validity of the proposed model with practical cases. Section 6 gives the conclusions of the whole paper and the prospects for future work.

## 2. Literature Review

The concept of group polarization was first proposed by American scholar Sunstein [6] in 1961, and he argued that if the views of group members had some bias at the beginning, this bias would be reinforced after discussion, and finally a consistent polarized view would be formed. At present, many scholars have studied the polarization of public opinion, and their research mainly includes two streams. One is research based on the single-dimension polarization of public opinion. The second is to expand the topic of network public opinion from one dimension to multiple dimensions and study the polarization process of multi-dimension public opinion.

At present, the research on unidimensional public opinion polarization is mainly analyzed from its internal and external influencing factors. The representative results are as follows. Chen et al. [7] studied the polarization phenomenon and establishes a public opinion polarization model with considerations of individual heterogeneity and dynamic conformity. Linde [8] revealed a positive relationship between party cues and perceptions of climate change risk, indicating a negative relationship between perceived polarization and individual risk perceptions. Das et al. [9] summarized that the two most important social influences opinion formation process were: (i) the majority influence caused by the existence of a large group of people sharing similar opinions, (ii) the expert influence originated from the presence of experts in a social group. Their model successfully captured opinion dynamics under the concomitant influence of the majority and the experts. In addition, by introducing social preference theory, Chen et al. [10] revealed the micro-interaction mechanism of public opinion polarization and pointed out that different social preferences held by individuals had different influences on public opinion polarization effects. Zhang [11] proposed a difference-driven model that suggested exposure to dissimilar views in democratic deliberation fosters reconsideration of policy preferences and that the mechanism of change varied by individual predispositions. Abeles et al. [12] studied the impacts of opinion deviance on global warming and found that people did not assume similarity with ingroups or dissimilarity with outgroups when a person’s own belief contrasted with the opinion cues sent by spokespeople and institutions affiliated with a person’s political party. Krause et al. [13] analyzed a voter model variant, where an additional undecided state of agents’ opinions was introduced. The model’s dynamics in the presence of strong repulsion led to a fifty-fifty stalemate where no opinion could win in the long run. Xiao et al. [14] thought that the evolution of individual opinions was not only influenced by the interactions between neighboring individuals but was also updated naturally due to individual factors themselves, in the absence of interaction. Chen et al. [15] suggested that individual internal characteristics and external intervention information affected public opinion reversal. When individual conservation was strong or individual attention was weak, even if external intervention information was strong, there would still be no obvious reversal of public opinion.

In addition, there are studies about the impacts of external factors on public opinion polarization. Some typical literature is as follows. Lee and Choi [16] found that network heterogeneity on social media could decrease polarization. The moderation effects of political orientation and fear of political opponents in the relationship between network heterogeneity and polarization were also found. Wojcieszak et al. [17] explored that strongly opinionated citizens exposed to news about the European Union (EU) polarized following exposure, and that the “easy” dimensions of EU attitudes polarized more than the “hard” attitude dimensions. These results extended the polarization literature to naturalistic settings and suggested that the polarizing effects of the media might be greater than previously acknowledged. Asker and Dinas [18] believed that online media could induce opinion polarization even among users exposed to ideologically heterogeneous views, by heightening the emotional intensity of the content. Higher affective intensity provoked motivated reasoning, which in turn led to opinion polarization. Sude et al. [19] considered that online contexts did not always foster polarization through selective exposure marked by a confirmation bias, and people’s attitudes were more moderate when they encountered inconsistent cues. Lin and Tian [20] studied an empirical case of how public debating on Weibo, China’s equivalent to Twitter, leads to opinion polarization. Weibo’s technical design, which enabled simultaneous interactions with multiple audiences (of which many users are unaware) further complicated the debate. Bolsen et al. [21] investigated an approach to communicating information about climate change that involved manipulating the source of a message, while holding the content of the message constant. The results revealed that messages attributed to military leaders, or to Republican Party leaders, could enhance the impact of the appeal. This finding underscored the importance that the source of any communication could have on its overall effectiveness. Gao et al. [22] introduced eight mechanisms, working on the formation and dissemination of public opinion on the network. Based on system dynamics, this article further proposed a comprehensive causal relationship model to explore the factors affecting the consequence of public opinion on the network. Particularly, the role of government was taken into consideration in this model. Gong et al. [23] concluded that, compared with opinion leaders, structural hole spanners had better locations in social networks to expand the scope of information diffusion. They also proposed a novel structural-hole-based approach to control public opinion in social networks.

The literature on the public opinion polarization phenomenon is mostly based on the single dimension of network public opinion topics, starting from the internal and external factors that affect public opinion polarization, and exploring the formation of the public opinion polarization process; although to a certain extent the study reveals the formation mechanism, it omits public opinion from the multidimensional discussion of the formation of polarization. With further disclosure of information related to the event, discussion topics of multiple dimensions will gradually emerge in the network, and individuals’ participation in topics of different dimensions will form their multidimensional attitudes, and the polarization of online public opinions will be promoted to present multi-dimensional characteristics through interaction with individual neighbors [24]. Due to the complexity of multi-dimensional topics, there are many influencing factors on the polarization process, but there is relatively little research. Based on all this, research on the multi-dimensional public opinion polarization phenomenon can reflect the formation process of public opinion polarization more comprehensively and objectively from the topic derived context.

At present, some scholars have made a preliminary discussion on the process of multi-dimensional public opinion polarization. By combining social judgment theory with the multi-agent model, Li and Xiao [25] proposed a multidimensional opinion evolution model for studying the dynamics of opinion polarization. The results demonstrated that polarization was influenced by the average degree of the network, and the polarization process was affected by the parameters of the assimilation effect and the contrast effect. Parsegov et al. [26] proposed a significant extension of the classical Friedkin-Johnsen model, describing the evolution of the agents’ opinions on several topics. Unlike the existing models, these topics were interdependent, and hence the opinions being formed on these topics were also mutually dependent. Wang et al. [27] built a multidimensional network model oriented toward the topology of public opinions on “the We Media” networks. The multidimensional network model could be used to effectively characterize the communication characteristics of multiple topics on “the We Media” networks. Although the above literature considers the multidimensional characteristics of online public opinion topics, they have also revealed the evolution mechanism of multidimensional public opinion to some extent. However, in the evolution process of individual multidimensional attitudes, only the influence of internal views on the same dimensional topic is considered, but the influence of the interaction between multidimensional topics and external intervention information is not considered. This deviates from the actual situation, which needs further study.

To sum up, most of the current research on the polarization of public opinion considers network public opinion from a single dimension, and merely studies multi-dimensional public opinion polarization from the perspective of topic derivation. In fact, in the evolution of online public opinion, the outbreak of a social hot event usually gives rise to discussion from multiple dimensions, and individuals’ participation in topics of different dimensions will form their multi-dimensional attitudes towards the event. At the same time, an individual’s attitude will change under the influence of internal and external aspects of the topic he or she participates in. Among these influences, internal influence comes from the views of neighbors within the same dimension of the topic, while external influence comes from the influence of other dimensions of the topic and external intervention information. Based on this, this paper first introduces the topic correlation coefficient and the influence intensity index of different dimensions of topics to describe the mutual influence among multi-dimensional topics, and defines the index of individual topic support, comprehensively reflecting the influence of different dimensions of topics and external intervention information on individual attitudes. Secondly, based on the model proposed by Jager and Amblard [28], a multi-dimensional attitude interaction rule is established to reflect the influence of neighbors’ views within the same dimensional topic, and a multi-dimensional public opinion polarization model in the topic-derived context is constructed. Finally, combined with the simulation experiment, the paper analyzes the influence of individual participation topic status, the degree of confidence in expressing an individual opinion, topic correlation coefficient, and external intervention information on multi-dimensional public opinion polarization, and verifies the rationality and effectiveness of the model through practical cases.

## 3. Model Construction

In this paper, modeling is carried out based on Monte Carlo the multi-agent method. Agents are used to represent individual nodes in the network. The network scale is set as N, i.e., there are N netizen nodes in the network. The individual’s initial attitude is (x_1_, x_2_, x_3_, …, x_n_), which is a point in n-dimensional space. The x_n_ belongs to the interval (0, 1), and x_n_ obeys N ~ (0.5, 0.2), mapping in the interval (0, 1). Set attitude (<0) as 0 and the attitude (>1) as 1. In this way, the initial attitude of most individuals is relatively neutral, while only a few individuals hold extreme attitudes. This assumption is consistent with the attitude distribution of groups in the real world towards certain kinds of events.

**Figure 1:**
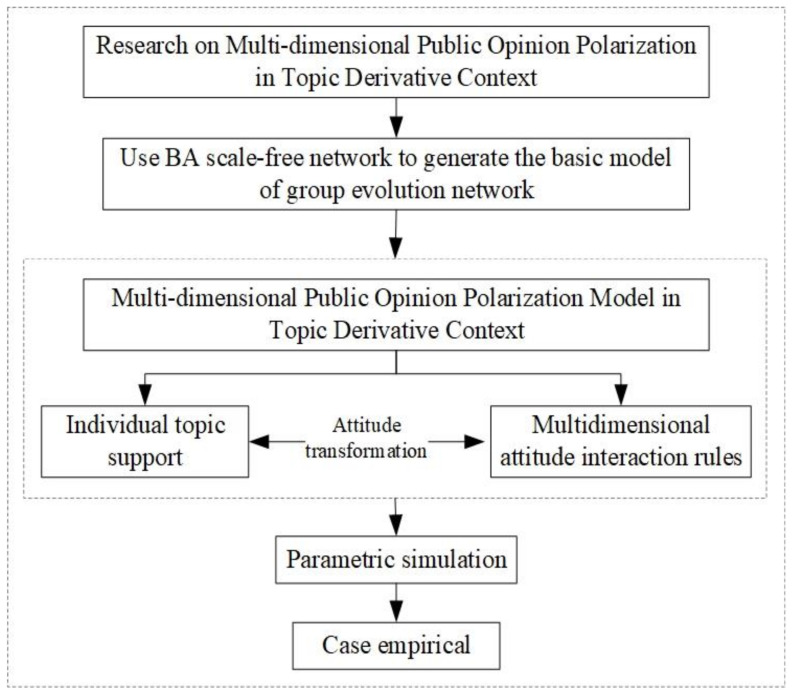
Research structure.

After the outbreak of social hot events, the related multiple information will spread rapidly in the network and gradually form multi-dimensional hot topics [29]. Under the influence of multiple information, individual *i* will often participate in the discussion of multiple related topics and form his own multidimensional attitude towards the event. In order to seek more consensus of views, individual *i* will further interact and communicate with individual neighbors. In this process, individual *i*’s attitude will be influenced by two aspects: one is the attitude interaction which means the exchange and communication of individual viewpoints between individuals from the same dimension; the other is the attitude interaction between individuals from a different dimension. These two factors affect the individual’s support degree, which further impacts on attitude tendency towards the topic. Based on this, this paper constructs a multi-dimensional public opinion polarization model in the topic derivative context. The research idea of this paper is shown in Figure 1:

As shown in Figure 1, this paper focuses on the problem of multidimensional opinion polarization in a topic-derived context. First, in order to simulate real social networks, this paper uses BA scale-free networks (proposed by Barabasi and Albert) [30] to generate a group evolutionary network. Second, by defining individual topic support indicators, the influence of external factors such as different dimensional topics and external intervention information on individual attitudes is integrated. Meanwhile, based on the idea of the J-A model, multidimensional attitude interaction rules among individuals are established to reflect the influence of neighbors’ opinions within the same dimensional topic. After that, a multidimensional opinion polarization model in topic-derived contexts is constructed. Finally, combined with the simulation experiment, the paper analyzes the influence of individual participation topic status, individual opinion confidence degree, topic correlation coefficient and external intervention information on multi-dimensional public opinion polarization, and verifies the rationality and effectiveness of the model through practical cases. The parameters and variables involved in the model are shown in Table 1 and Table 2.

**Figure 2:**
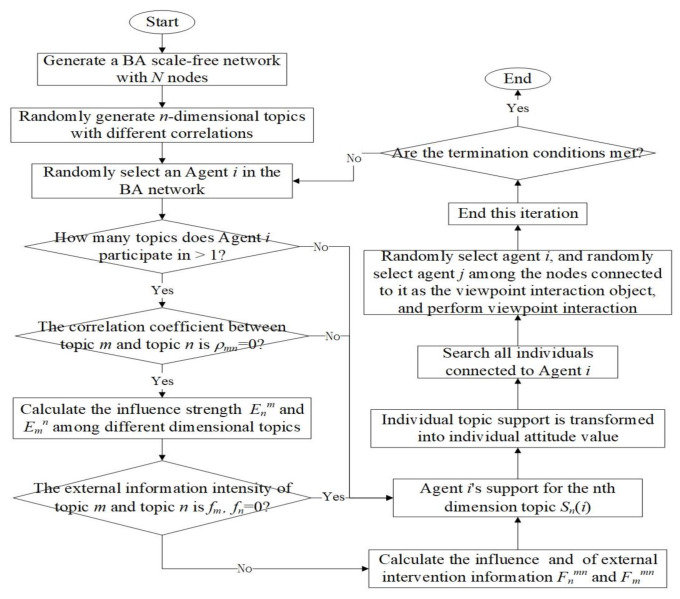
Simulation schematic diagram based on Multi-Agent Monte Carlo approach.

Based on the above analysis, the simulation process of this paper is shown in Figure 2:

As shown in Figure 2, this paper simulates the above proposed multidimensional opinion polarization model based on the multi-agent approach of Monte Carlo, and the specific process is as follows:

Step1: Build the initial network: the initial network nodes are *m*_0_, and the nodes are connected randomly.

Step2: The growth of network nodes: *m*_1_ new nodes are added to the network each time, and a connection is established with *m*_0_ nodes in the initial network, that is, *m*_1_ new edges are added each time. The connection probability of the newly added nodes connecting the nodes in the initial network is positively correlated with the original node degree, and the resulting undirected network graph with node size *N* is generated.

Step3: Randomly generate n-dimensional topics with different topic correlation coefficients.

Step4: Randomly select an Agent *i* in the generated network and determine whether the number of topics it participates in is greater than 1. If “yes”, Step5 is executed, otherwise Step9 is executed.

Step5: Determine whether the correlation coefficient *ρ_mn_* of topic *m* and topic *n* is equal to 0. If “No”, then Step6 is executed, otherwise Step9 is executed.

Step6: Calculate the influence strength $E_{n}^{m}$ and $E_{m}^{n}$ among different dimensional topics according to Equations (3) and (4).

Step7: Determine whether the information intensity *f_m_* and *f_n_* of topic *m* and topic *n* is 0, if “No” then execute Step 8, otherwise execute Step9.

Step8: Calculate the influence $F_{n}^{mn}$ and $F_{m}^{mn}$ of external intervention information according to Equations (5) and (6), and continue with Step9.

Step9: Update the topic support and attitude values of Agent *i* according to the rules, and continue to execute Step10.

Step10: Randomly select agent *i*, and randomly select agent *j* among the nodes connected to it as the viewpoint interaction object and perform viewpoint interaction according to Equations (7)–(12).

Step11: Repeat step4–10 until the end of the evolution time.

### 3.1. Individual’s Support Degree of the nth Dimensional Topic $S_{n}\left(i\right)$

Because of the differences in social background and the style of thinking, even when facing the same social hot event, individuals have different attention and acceptance for different dimensional topics [31]. The attention and acknowledgment of different topics are defined as $S_{n}\left(i\right)$, indicating the attitude value of individual *i* to the *n*th dimensional topic. On the one hand, it is affected by the individual’s attitude value towards the *n*th dimensional topic; On the other hand, it will be influenced by other dimensional topics and external intervention information. Based on this, its expression is as follows (Equation (1)):

(1) $$S_{n}(i)=\;x_{n}\left(i\right)+\left(1-C\left(i\right)\right)*E_{n}^{m}+F_{n}^{mn}$$

where $F_{n}^{mn}$ represents the comprehensive influence of external intervention information on the *n*th dimensional topic.

The impact of topic m on topic *n* will be affected by *C*(*i*) (*C*(*i*)∈(0, 1)), the opinion confidence of individual *i*. The larger value of *C*(*i*) indicates that the individuals are more confident and less likely to be affected by other dimensional topics.

With the change of individual topic support, their attitude value will also change. Generally speaking, the higher an individual’s support for a certain dimension of the topic, the greater his attitude towards that topic will be. Based on this, the conversion rules between an individual’s support degree and an individual attitude value are defined as follows (Equation (2)):

(2) $${\dot{x}}_{n}\left(i\right)=\left\{\begin{array}{c} 1,\;\;\;\;S_{n}(i)>1\\ S_{n}(i),0⩽S_{n}\left(i\right)⩽1\\ 0,\;other \end{array}\right.$$

where the larger the value of $S_{n}\left(i\right)$ is, the greater its attitude value will be correspondingly. When $S_{n}\left(i\right)$ is less than 0, it means that the individual does not support the *n*th dimensional topic, so its attitude value is 0.

#### 3.1.1. Topic Correlation Coefficient *ρ_mn_*

For multi-dimensional topics derived from the same event, there is usually a relatively complex relationship among them, which will have an important impact on the evolution process of multi-dimensional public opinions. Therefore, this paper refers to the Pearson correlation coefficient [32] to describe statistically the complex relationships among topics of different dimensions. In combination with Pearson’s correlation coefficient, this paper summarizes the relationships among topics of different dimensions into the following categories. Specific rules are shown in Table 3:

#### 3.1.2. Influence Intensity of Different Dimensional Topic $E_{n}^{m}$

As hot events continue to ferment, multi-dimensional topics derived from them will form one social circle after another, and different social circles will influence and compete with each other in order to obtain more opinions, thus causing the heat of various topics in the public opinion field to ebb and flow [33]. Usually, the interaction among topics in different dimensions involves two factors: on the one hand, the number of participants and their comprehensive attitude value towards a topic in a certain dimension, which represents the comprehensive influence of the topic; on the other hand, the topic correlation coefficient including correlation and correlation degree, in which correlation determines the direction of mutual influence between topics, while correlation degree determines the degree of mutual influence between topics. In combination with the topic correlation coefficient, the rules of interaction between topics are defined, as shown in Equations (3) and (4):

(3) $$e_{n}^{m}=\frac{\sum x_{m}\left(i\right)}{\sum_{S_{n}\left(i\right)>0}i}\left(m=1,2,3\ldots ;n=1,2,3\ldots \right)$$

where $e_{n}^{m}$ represents the impact of the *m*th dimensional topic on the nth dimensional topic, which is affected by total group attitude for the *m*th dimensional topic and the number of individuals taking part in the *n*th dimensional topic. The ratio of these two represents the average influence of the comprehensive attitude tendency of the *m*th dimensional topic on the individuals participating in the *n*th dimensional topic, which can reflect the influence degree of the *m*th dimensional topic on the *n*th dimensional topic.

(4) $$E_{n}^{m}=\left\{\begin{array}{c} \rho_{mn}*e_{n}^{m},\;\;\;\;0⩽e_{n}^{m}⩽1\\ \rho_{mn}*1,\;\;\;\;other \end{array}\right.$$

where $E_{n}^{m}$ represents the intensity of the impact of the *m*th dimensional topic on the *n*th dimensional topic ($E_{n}^{m}$ ∈ (−1, 1)), which is affected by $e_{n}^{m}$ and *ρ_mn_*. When *ρ_mn_* > 0, the *m*th dimensional topic has a positive influence on the *n*th dimensional topic. When $e_{n}^{m}$ > 1, it means that the influence of the *m*th dimensional topic is much greater than that of the *n*th dimensional topic. For convenience, the default maximum value of $e_{n}^{m}$ is 1.

#### 3.1.3. The Influence of External Intervention Information $F_{n}^{mn}$

In the process of derivation and spread of multi-dimensional topics, external intervention information has always played an important role [34]. In fact, external information about any dimensional topic can have an impact not only on itself, but also on its related topic. In general, the stronger the external intervention information of any dimensional topic is, the stronger the influence on that topic and its related topics will be. Based on this, the influence rules of external intervention information are defined in combination with the topic correlation coefficient, as shown in Equations (5) and (6).

(5) $$F_{n}^{m}=\rho_{mn}*\sin(\frac{\pi }{2}*f_{m})$$

where $F_{n}^{m}$ represents the influence of the impact of external intervention information of the *m*th dimensional topic on the *n*th dimensional topic, which is affected by *ρ_mn_*, and *f_m_*. *f_m_* > 0 represents the external positive information of the *m*th dimensional topic, while *f_m_* < 0 represents the external negative information of the *m*th dimensional topic. $sin(\frac{\pi }{2}*f_{m})$ is increasing in the interval [−1, 1] and the domain is [−1, 1], whose changing trend is consistent with the influence of external invention information on an individual’s support. When *f_m_* < 0, there is a negative correlation between topic m and topic *n*. At this point, the external information of the *m*th dimensional topic will have a completely opposite influence on the *n*th dimensional topic.

(6) $$F_{n}^{mn}=\sin(\frac{\pi }{2}*f_{n})+F_{n}^{m}$$

where $F_{n}^{mn}$ represents the comprehensive influence of external intervention information on the *n*th dimensional topic, which is not only affected by the intensity of external information on the *n*th dimensional topic, but also affected by the external intervention information on related topics.

### 3.2. Interaction Rule of Multidimensional Attitude

Due to the social characteristics of Internet users, when they are interested in multi-dimensional topics derived from a hot event and form opinions, in order to reach consensus with more Internet users, they will choose to exchange views with individual neighbors and constantly update their own attitudes [35]. Therefore, based on the viewpoint interaction idea of the J-A model, this paper extends this from one dimension to multi-dimensions, and constructs multi-dimensional attitude interaction rules.

In the process of individual interaction, an individual updates his own attitude value by comparing it with the attitude value $x_{j}^{n}$ of its neighbor nodes. When the attitude values of individuals and neighbor nodes are similar (in the assimilation effect zone), individuals tend to be closer to the attitude of neighbor nodes. When there is a significant difference in attitude value between individuals and their neighbors (in the repulsion zone), individuals tend to enlarge the difference in attitude. The specific rules are as follows (Equations (7)–(12)):

1. when $\sqrt{\sum {\left({\dot{x}}_{n}\left(i\right)-{\dot{x}}_{n}\left(j\right)\right)}^{2}}<d_{1}$
  (7) $${\ddot{x}}_{n}\left(i\right)={\dot{x}}_{n}\left(i\right)+\mu \left({\dot{x}}_{n}\left(j\right)-{\dot{x}}_{n}\left(i\right)\right)$$
  (8) $${\ddot{x}}_{n}\left(j\right)={\dot{x}}_{n}\left(j\right)+\mu \left({\dot{x}}_{n}\left(i\right)-{\dot{x}}_{n}\left(j\right)\right)$$
  where *n* = 1, 2, 3, …, *μ* ∈ (0,0.5]. *μ* is called the convergence parameter.
2. when $\sqrt{\sum {\left({\dot{x}}_{n}\left(i\right)-{\dot{x}}_{n}\left(j\right)\right)}^{2}}>d_{2}$
  (9) $${\ddot{x}}_{n}\left(i\right)={\dot{x}}_{n}\left(i\right)-\beta \left({\dot{x}}_{n}\left(j\right)-{\dot{x}}_{n}\left(i\right)\right)$$
  (10) $${\ddot{x}}_{n}\left(j\right)={\dot{x}}_{n}\left(j\right)-\beta \left({\dot{x}}_{n}\left(i\right)-{\dot{x}}_{n}\left(j\right)\right)$$
  where *n* = 1, 2, 3, …, *β* > 0. *β* is called the divergent parameters.
3. In other cases, the attitude values of individual *i* and *j* remain unchanged, which is expressed as follows:
  (11) $${\ddot{x}}_{n}\left(i\right)={\dot{x}}_{n}\left(i\right)$$
  (12) $${\ddot{x}}_{n}\left(j\right)={\dot{x}}_{n}\left(j\right)$$

## 4. Simulation Experiment

Combined with the multi-dimensional public opinion polarization model constructed above, this section discusses the influence of individual participation topic status, individual opinion confidence and interaction times, topic correlation coefficient and external intervention information on the multi-dimensional public opinion polarization phenomenon, and further reveals its internal evolutionary mechanism.

### 4.1. The Influence of Individual Participation Topic Status on the Multi-Dimensional Public Opinion Polarization Process

Combined with the multi-dimensional public opinion polarization model mentioned above, the initial parameters of model evolution are set. In order to describe the distribution of extreme attitude, individuals with attitude values greater than 0.9 are called strongly positive individuals, individuals with attitude value less than 0.1 are called strongly negative individuals, and the proportion of extreme individuals in the group is called the polarization rate. This can be divided into *S* polarization and *O* polarization rate: the former represents the proportion of extreme positive individuals in the group, and the latter represents the proportion of extreme negative individuals in the group. In order to simplify the process, this article only studies S polarization rate. The BA scale-free network was selected for the simulation network, and the node size was set as 500. Taking visualization for full consideration other parameters are set as: *d*_1_ = 0.3, *d*_2_ = 0.55, *μ* = 0.25, *β* = 0.1, the opinion confidence *C* of individual *i* obeys N~(0.5, 0.2), mapping in the interval [0, 1]. In order to observe the evolution of multi-dimensional public opinions, the correlation of topics in different dimensions is set to be strongly negative, with the evolution time *T* = 50. In this paper, the evolution results of three-dimensional topics are taken as an example for analysis, and the conclusion can be extended to other multi-dimensional situations.

#### 4.1.1. The Influence of Individual Participation in Different Number of Topics on the Multi-Dimensional Public Opinion Polarization Process

**Figure 3:**
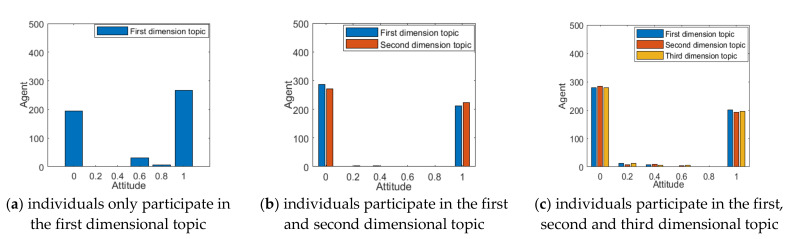
The distribution of group attitude when individuals participate in different topics.

**Figure 4:**
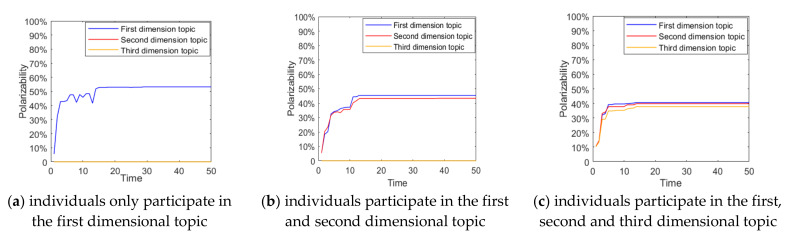
The polarizability of group attitude when individuals participate in different topics.

When a multi-dimensional topic is derived from a hot event, individuals tend to participate in part or full in the discussion according to their interests and cognition. The number of different individuals participating in the topic discussion will be different, and this difference in selection will impact on the multi-dimensional public opinion polarization. Based on this, this section will analyze the situations in which individuals participate in discussion on different numbers of topics, and the results are shown in Figure 3 and Figure 4.

As can be seen from Figure 3 and Figure 4, when individuals only participate in the first dimensional topic, most individuals’ attitude values gather around 1, and the polarization rate is around 50%, indicating that there is only one topic diffusion in the public opinion field at this time, and a strong public opinion polarization phenomenon is formed. As the number of topics that individuals participate in increases, due to the strong negative correlation between the different dimensions of the topic, the attitude value of most individuals gathers towards 0, and the corresponding polarization rate also decreases. This indicates that with the increase in the number of individuals participating in the topic discussion, the conflict between topics will make some individuals’ attitudes more relaxed, and the effect of public opinion polarization will also decline.

**Figure 5:**
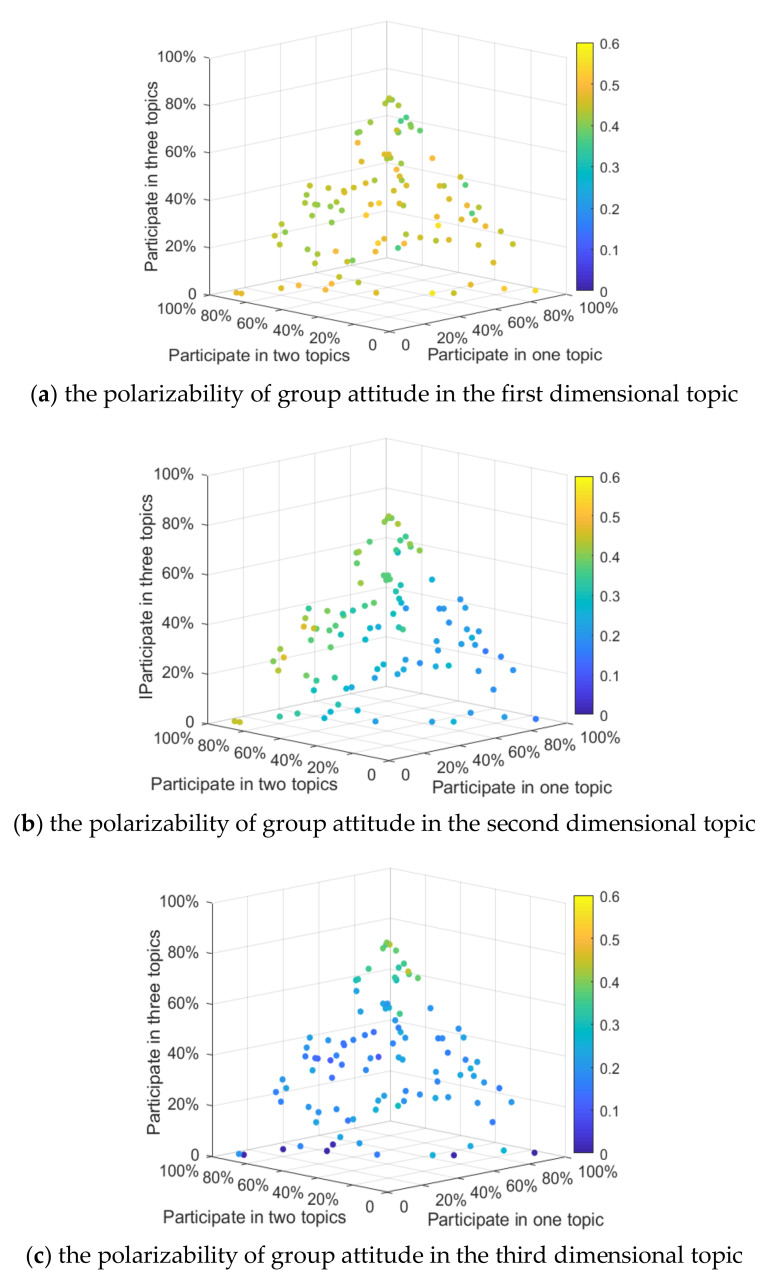
The polarizability of group attitude when individuals randomly engage in several topics.

In order to further analyze the influence of the number of individuals participating in the topic discussion on multi-dimensional public opinion polarization, based on the above analysis, a form of random distribution is designed. To facilitate the control of variables, the following rules are made: (1) when individuals only participate in the discussion of one topic, it is assumed that the individual participates in the topic in dimension 1; (2) when individuals participate in the discussion of two topics, it is assumed that they participate in the topics in dimension 1 and 2; (3) When individuals participate in the discussion of three topics, it is assumed that they participate in the topics in dimension 1, 2 and 3. 100 times tests are carried out respectively; in each test, a different topic number of individuals is randomly generated and assembled into different social networks (e.g., the proportion participating in discussion of one topic accounts for 45%, of two topics 28%, and of three topics 27%, respectively). The proportion rate of each combination is recorded and a 4D scatter plot (color as a dimension of a four-dimensional scatter plot is used to describe the change in attitude polarizability) is drawn. The results are shown in Figure 5:

It can be seen from Figure 5a that, with the increase in the proportion of individuals participating in a topic discussion, the attitude polarization rate of topics in the first dimension does not change significantly. This is because when rules were set above, individuals would always participate in the discussion of topics in the first dimension, so their attitude polarization rate does not change significantly. As can be seen from Figure 5b, as more individuals choose to participate in the discussion of two or more topics, the attitude polarization rate of the second dimensional topics increases continuously. At the same time, Figure 5c shows that when the number of individuals involved in three topics account for more than 60%, the third dimensional topic attitude polarization rate is significantly enhanced. Contrasting Figure 5c with Figure 5a,b, it can be found that, as the number of people participating in three topics increases, attitude polarizability at this time is around 50%. The reason is that as the number of people participating in three topics increases, the dimension subject participation gradually converges, and the influence of the different dimensions becomes the same, so the attitude polarizability for all dimensional topics gradually becomes the same. This indicates that, with the increase in the number of participants in any dimension, the influence of the topic will be enhanced accordingly, thus increasing the effect of public opinion polarization. At the same time, when the number of participants in each dimension is relatively consistent, the effect of public opinion polarization regarding the topic in each dimension gradually tends to be consistent.

#### 4.1.2. The Dynamic Influence of Opinion Leaders and Topic Correlation Coefficient on the Number of Topics that Individuals Participate in

**Figure 6:**
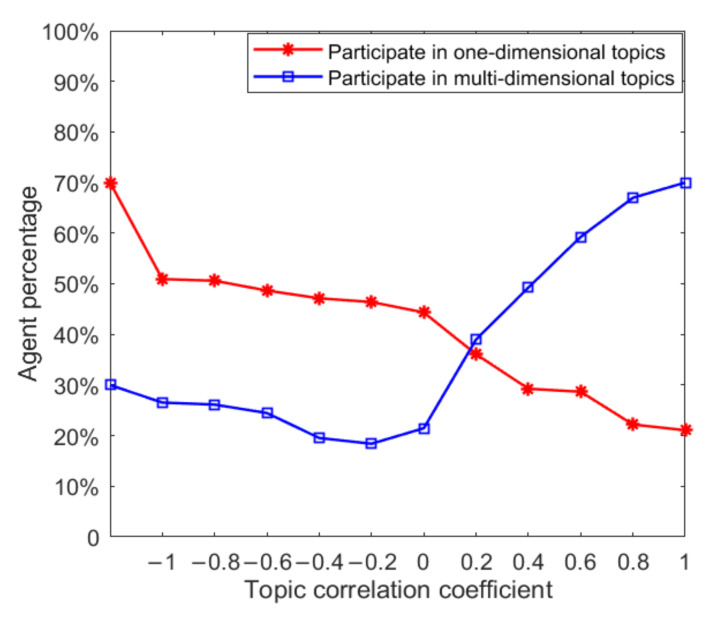
The influence of opinion leaders on the number of topics that individuals participate in under different topic correlation coefficients.

In the process of multi-dimensional public opinion evolution, individuals’ participation in derivative topics will change dynamically under the influence of opinion leaders and related topics. This section will study the dynamic changes in the number of topics that individuals participate in. First, this paper searched for opinion leaders according to the concept of degree in a social network, calculated the degree of each node and ranked it, and selected opinion leaders from high to low. Because opinion leaders have advantages in thinking mode and information acquisition, it is assumed that opinion leaders will participate in the discussion of three topics at the initial moment, while ordinary individuals only participate in the discussion of one-dimensional topics. In the process of public opinion evolution, ordinary individuals under the influence of opinion leaders will choose to participate in discussions of more dimensional topics, so as to observe the influence of opinion leaders on the number of topics that ordinary individuals participate in. To facilitate the control of variables, 30% of the nodes are selected as opinion leaders, and 70% of the nodes are ordinary individuals (i.e., in the initial state, 70% of individuals participate in the discussion of one topic and 30% participate in the discussion of three topics). The results are shown in Figure 6.

In Figure 6, the red curve represents the proportion of individuals taking part in one dimensional topic discussion. Compared to its initial state, under the influence of opinion leaders and with the augmentation of the topic correlation coefficient, the proportion continues to decline, which indicates that, with the inclusion of a certain proportion of opinion leaders, more individuals choose to participate in discussion of various topics. With the augmentation of the topic correlation coefficient, the individual is more willing to participate in discussion of various topics. The blue curve represents the proportion of individuals participating in the discussion of multi-dimensional topics, indicating that when the topic correlation coefficient is greater than 0compared with the initial state, the proportion of individuals participating in the discussion of multi-dimensional topics increases with the enhancement of the topic correlation, and vice versa. This shows that when there is a negative correlation between multi-dimensional topics, the addition of a certain proportion of opinion leaders in the network will not make more individuals participate in the discussion of multi-dimensional topics. However, when there is a positive correlation between multi-dimensional topics, the participation of a certain proportion of opinion leaders can promote more individuals to participate in the discussion of multi-dimensional topics. Therefore, under public opinion control, when the direction of public opinion of the derived topic is consistent with that of the original topic, it is likely to lead to a wider range of public opinion polarization.

**Figure 7:**
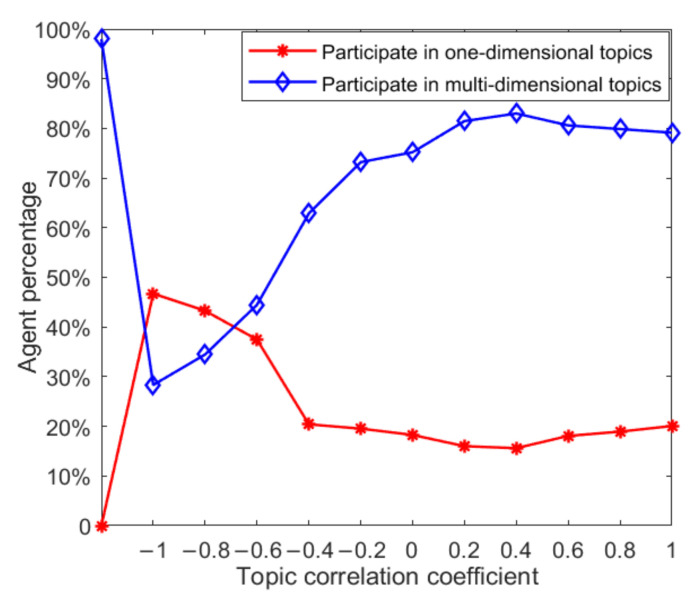
The influence of topic correlation coefficient on the number of topics that individuals participate in.

The above analysis can explain the factors affecting the change in individuals from participating in the discussion of single dimensional topics to multi-dimensional topics. The following will further analyze the factors affecting this change in combination with the topic correlation coefficient. Under the initial conditions, all individuals were set to participate in the discussion of topics in three dimensions, and the number of topics that individuals participated in was observed to change with the topic correlation coefficient. The results are shown in Figure 7.

As can be seen from Figure 7, compared with the initial state, with the progress of individual interaction and the interaction between different dimensional topics, the proportion of the number of individuals participating in multi-dimensional topic discussion decreases, while the proportion of the number of individuals participating in one dimensional topic discussion increases. The blue curve in the figure represents the proportion of individuals participating in the discussion of multi-dimensional topics. It can be seen that the proportion of individuals participating in the discussion of multi-dimensional topics increases with the increase of the topic correlation coefficient, and when the topic correlation coefficient is less than 0, the proportion of the number of individuals increases further. This shows that when multi-dimensional topics are negatively correlated under the mutual influence of different dimensional topics, individuals will choose to give up or reduce their support for a certain dimensional topic, so that the proportion of individuals participating in multi-dimensional topic discussion keeps decreasing. This is basically consistent with the reality, i.e., when there is a serious conflict of public opinions between multi-dimensional topics, netizens will gradually give up their participation in a certain dimensional topic according to their own judgment.

### 4.2. The Influence of Individual Opinion Confidence and Interaction Times on the Multi-Dimensional Public Opinion Polarization Process

The interaction among the topics of each dimension will have an important influence on the multi-dimensional public opinion polarization process, while individual factors will reduce or enlarge the effect between topics, and then have an influence on the multi-dimensional public opinion polarization process [36]. Therefore, the following will study the inhibiting or promoting effect of individual factors on the influence of different dimensional topics from two aspects, and then analyze its influence on the multi-dimensional public opinion polarization process.

#### 4.2.1. The Influence of Individual Viewpoint Confidence on the Multi-Dimensional Public Opinion Polarization Process

**Figure 8:**
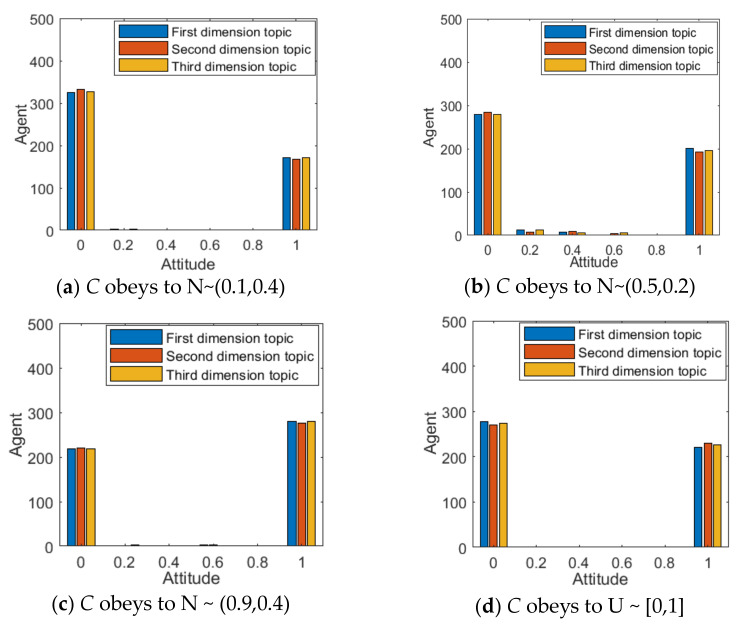
Histogram of group attitude distribution under individual view confidence with different distributions.

**Figure 9:**
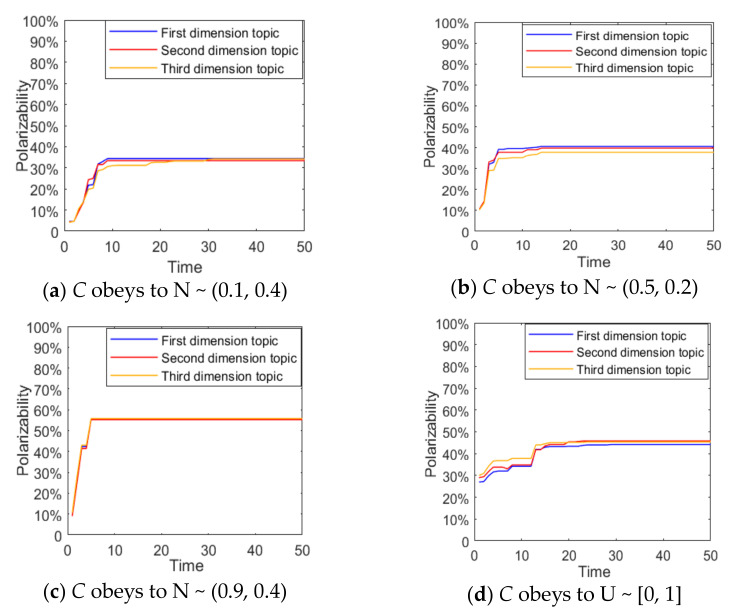
Polarizability curves of group attitude under individual view confidence with different distributions.

In order to describe the confidence of different opinions held by different individuals, the confidence of individual opinions C is subject to N~(0.1,0.4) and mapped to (0,1), indicating that the individual viewpoint confidence is generally low, that is, it is easy to be affected by other dimensional topics. C is subject to N~(0.9,0.4) and mapped to (0,1), indicating a generally high level of individual viewpoint confidence. C is subject to N~(0.5,0.2) and mapped to (0,1), indicating a general level of individual viewpoint confidence. C is subject to U~(0,1) to describe the situation where the number of individuals with different individual viewpoint confidence is roughly the same in the network. At this point, it is set that all individuals participate in the discussion of three dimensional topics, and the results are shown in Figure 8 and Figure 9.

From Figure 8 and Figure 9, when *C* is subjected to N ~ (0.1, 0.4), the distribution of group attitudes of various dimensions presents the polarization state, and the number of individual attitude values around 0 is significantly higher than those around 1. Meanwhile, the corresponding attitude polarization is around 35%, suggesting that when *C* is low, individuals are easily influenced by other dimensional topics, and thus weaken their own attitude. However, when *C* is subject to N ~ (0.9,0.4), individuals’ opinion confidence is generally high, and individuals are not easily affected by other dimensional topics. Therefore, the distribution of group attitude is around 1, and the polarization rate is around 55%, which is significantly higher. When the individuals’ viewpoint confidence is relatively general or more evenly distributed, that is, when *C* is subject to N ~ (0.5,0.4) and U ~ [0, 1], their attitude polarization rate increases compared with that of individuals with low confidence. Therefore, it can be concluded that when there is a negative correlation between multi-dimensional topics, with the increase of the confidence of an individual’s point of view, its restraining effect on the influence of other dimensional topics will be enhanced, thus promoting the effect of multi-dimensional public opinion polarization, and vice versa.

#### 4.2.2. The Influence of Individual Interaction Times on the Multi-Dimensional Public Opinion Polarization Process

**Figure 10:**
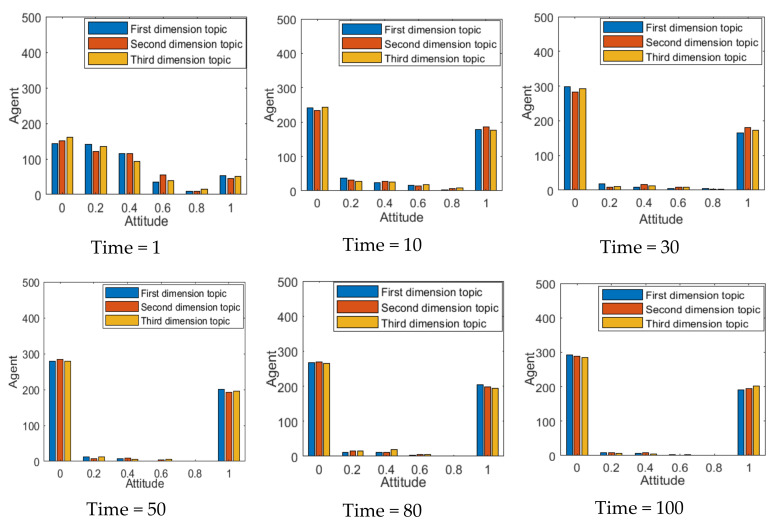
Histogram of group attitude distribution under different interaction times.

As an important part of individual attitude renewal, the individual interaction process plays an important role in the formation and polarization of multi-dimensional public opinion. The number of interactions between individuals is usually regarded as the depth of their opinion communication, and the deeper the communication between individuals, the easier it is to reach consensus, which is of great significance for the formation of multi-dimensional public opinion polarization. The results are shown in Figure 10.

As can be seen from Figure 10, when the number of individual interactions is 1, there is no in-depth point of view communication among individuals, so the distribution of group attitudes is relatively scattered and no obvious public opinion polarization phenomenon is formed. With the increase of the number of individual interactions, the number of individuals with a neutral attitude gradually decreases, and the group attitude forms an obvious polarization phenomenon. When the number of interactions reaches 50, the distribution of the group attitude gradually tends to be stable. However, due to the negative correlation between multi-dimensional topics, the distribution of group attitude is more inclined to the side of attitude value 0 under the influence of other dimensional topics. This shows that the number of individual interactions can affect the outcomes of multi-dimensional public opinion polarization and, with the deepening of individual interactions, attitude polarization tends to be steady.

**Figure 11:**
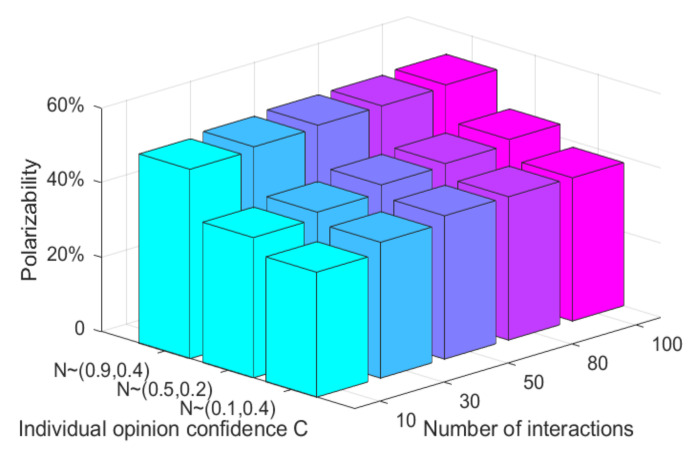
The relationship of individual viewpoint confidence, individual interaction times and attitude polarizability.

From the above analysis, it can be seen that both individual opinion confidence and individual interaction times have an impact on the effect of multi-dimensional public opinion polarization. As two important factors affecting public opinion polarization at the individual level, it will be further analyzed in the form of a combination of factors. Here, only the evolution results of the first dimensional topic are analyzed, and the results of other dimensions are basically consistent with them, as shown in Figure 11.

As can be seen from Figure 11, with the gradual increment of C, the attitude polarization rate has a relatively obvious increment, while with the increment of the number of individual interactions, the attitude polarization rate has a relatively small change. This indicates that, compared with the number of individual interactions, individual opinion confidence has a greater impact on the polarization effect of multi-dimensional public opinions. Moreover, with the increment of individual opinion confidence, the polarization effect of public opinions on all dimensional topics is stronger.

### 4.3. The Influence of Topic Correlation Coefficient on the Multi-Dimensional Public Opinion Polarization Process

The topic correlation coefficient will have an impact on the interaction between topics of different dimensions, which includes two aspects: first, the topic correlation, which affects the direction of the interaction between them; second, the degree of topic correlation, which affects the intensity of interaction between each other. The following will analyze the influence of the topic correlation coefficient on the multi-dimensional public opinion polarization process.

#### 4.3.1. The Influence of Topic Correlation on the Multi-Dimensional Public Opinion Polarization Process

**Figure 12:**
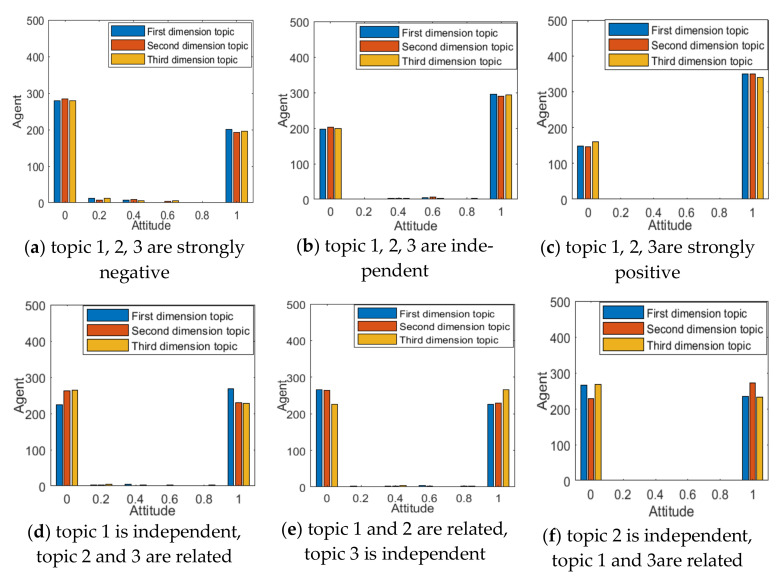
Histogram of group attitude distribution under different topic correlations.

**Figure 13:**
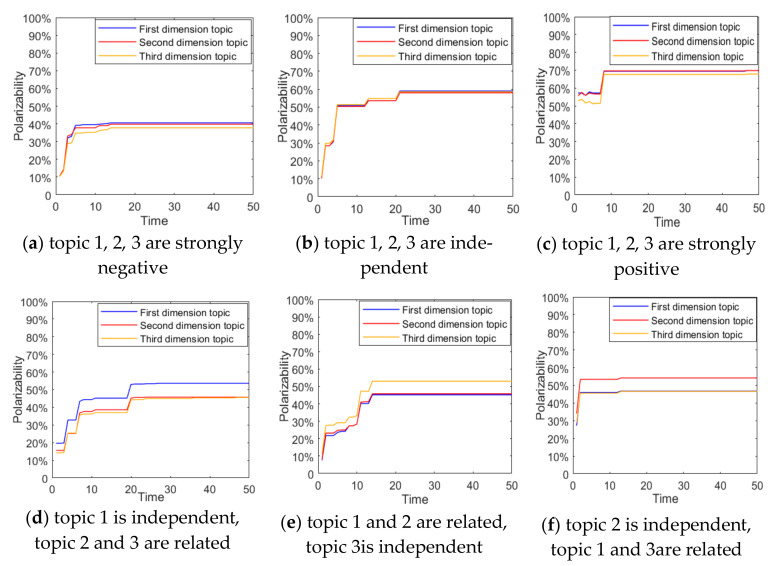
The polarizability curves of group attitudes under different topic correlations.

In order to analyze the interaction mechanism between different dimensional topics, this section will combine the concept of topic correlation coefficient defined above to analyze the influence of topic correlation on multi-dimensional public opinion polarization [37]. To facilitate the control of parameters, when there is only topic correlation of any two dimensions, the default relationship is a strongly negative correlation. The results are shown in Figure 12 and Figure 13.

It can be seen from Figure 12a that, when three-dimensional topics are negatively correlated, the interaction among topics in different dimensions makes the distribution of group attitude values more inclined to the 0 side. This shows that when the conflict of public opinions between multi-dimensional topics is serious, some individuals will give up their support for a certain dimensional topic. The opposite was true when the three-dimensional topics were positively correlated. At the same time, it can also be found that when the three-dimensional topics are all positively correlated, the corresponding attitude polarizability is the largest, followed by mutual independence, and the negative correlation is the lowest. This shows that the correlation between multi-dimensional topics will have an important impact on polarization. When there is a positive correlation between multi-dimensional topics, the effect of public opinion polarization will be enhanced. When there is negative correlation between multi-dimensional topics, the effect of public opinion polarization will be weakened. Therefore, in public opinion control, attention should be paid to the relationship between multi-dimensional topics derived from a certain event. When the direction of public opinion among multi-dimensional topics is relatively consistent, it is likely to form the phenomenon of public opinion resonance. At the same time, correct information should be published in a timely manner in response to distorted information in order to weaken the phenomenon of multi-dimensional public opinion polarization.

At the same time, it can be seen from Figure 12 and Figure 13 that when there is a negative correlation between any two-dimensional topics, they will also have a mutual influence on each other. However, compared with three-dimensional topics, since there are only two topics influencing each other at this time, topics in each dimension are less influenced by other topics, so polarizability is relatively high. It also shows that the degree of interaction between multi-dimensional topics is not only affected by the topic relevance, but also by the number of related topics.

#### 4.3.2. The Influence of Topic Correlation on the Multi-Dimensional Public Opinion Polarization Process

**Figure 14:**
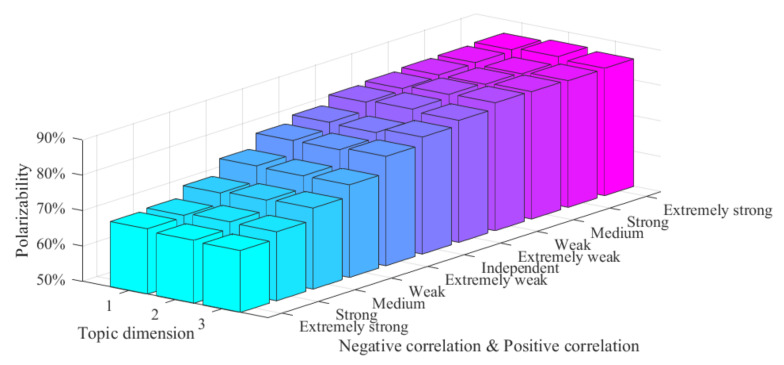
The polarizability curves of group attitudes under different topic correlations.

When there is a certain correlation between multi-dimensional topics, they will influence each other and enhance or weaken the effect of multi-dimensional public opinion polarization. However, the degree of mutual influence among topics of different dimensions will be influenced by the degree of topic correlation. The stronger the degree of correlation, the stronger the mutual influence will be. The following will analyze the influence of topic correlation degree on the multi-dimensional public opinion polarization process, and the results are shown in Figure 14.

In Figure 14, *x*-axis represents the degree of correlation between topics in different dimensions, which can be divided into negative correlation (extremely strong, strong, medium, weak, extremely weak), mutual independence, and positive correlation (extremely strong, strong, medium, weak, extremely weak), *y*-axis represents the dimension of topics (1, 2, 3), and *z*-axis represents the polarizability. It can be seen from Figure 14 that, when multi-dimensional topics are negatively correlated, the polarizability of each topic dimension increases gradually with the decrease of the degree of topic correlation. However, when the multi-dimensional topics are positively correlated, the polarizability of each dimension gradually increases and tends to be steady with the increase of the degree of topic correlation. This indicates that the degree of topic correlation will have an impact on the effect of multi-dimensional public opinion polarization. However, when the public opinion direction of multi-dimensional topics is relatively consistent, the mutual influence between them gradually tends to be stable, and the polarizability is stable at a maximum.

**Figure 15:**
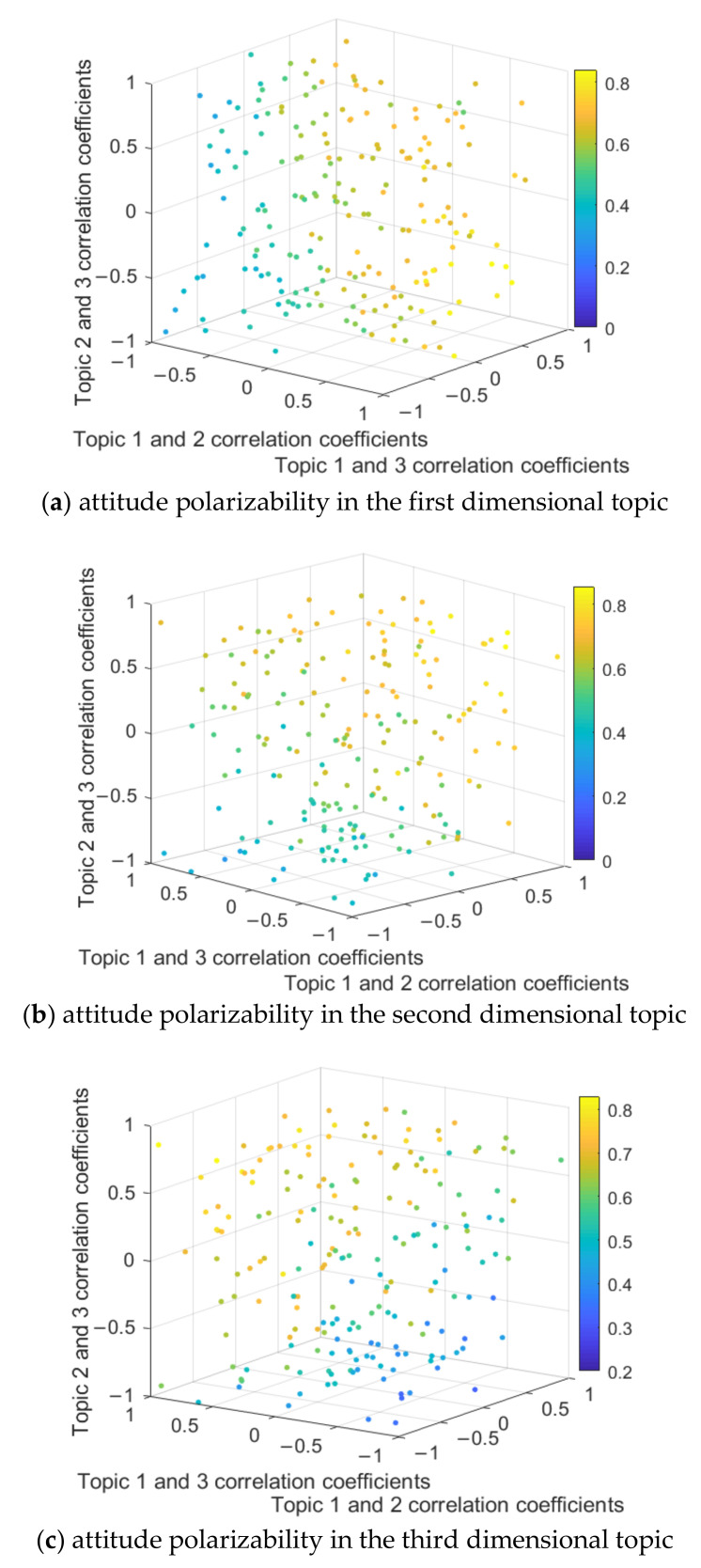
Attitude polarizability when the topic correlation coefficients are randomly distributed.

In order to comprehensively analyze the role of the topic correlation coefficient in the process of multi-dimensional public opinion polarization, the following content sets the correlation coefficient between topics in different dimensions through the form of random distribution and observes the change of topic polarizability in each dimension. 200 trials were conducted respectively, and each trial randomly generated the correlation coefficient between two topics (e.g., the correlation coefficient between topic 1 and topic 2 is −0.2; the correlation coefficient of topic 1 and 3 is 0.4; The correlation coefficient of topic 2 and 3 is 0.6). The polarizability of each combination is recorded, and a four-dimensional scatter plot of the polarizability is drawn, (Color as a dimension of a four-dimensional scatter plot describes the change in the attitude polarizability). The results are shown in Figure 15.

The Figure 15a shows that when the correlation coefficient between topic 1 and topic 2, 3, is greater than 0 (a positive correlation), the attitude polarizability of topic 1 is relatively high, but when the correlation coefficient between topic 1 and topic 2, 3, is less than zero, the attitude polarizability of topic 1 is relatively low. At the same time, as the correlation coefficient between topic 2 and 3 changes, the attitude polarizability of topic 1 does not significantly change, which is also shown in Figure 15b,c. This indicates that when any dimensional topic is positively correlated with other dimensional topics, the attitude polarizability of this dimensional topic is relatively high, and vice versa.

### 4.4. The Influence of External Intervention Information on the the Multi-Dimensional Public Opinion Polarization Process

After the outbreak of a hot event, with the release of external intervention information in different dimensions, individuals’ attitudes towards topics in different dimensions will change accordingly, which will lead to the change of public opinion polarization direction and the extension of the public opinion evolution process. Therefore, this section will comprehensively analyze the influence of external intervention information on the multi-dimensional public opinion polarization process.

#### 4.4.1. The Influence of External Intervention Information on the Topic Derivation Process

**Figure 16:**
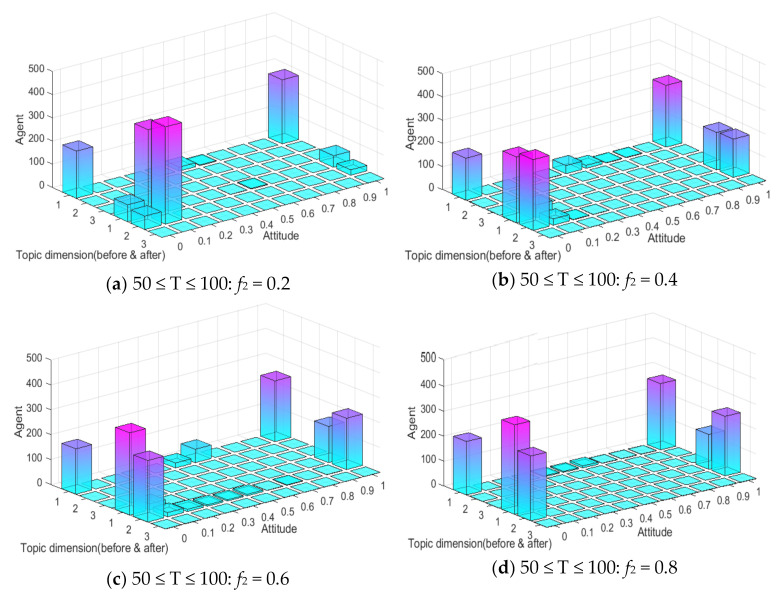
Histogram of group attitude distribution under different external information intensity at different moments.

**Figure 17:**
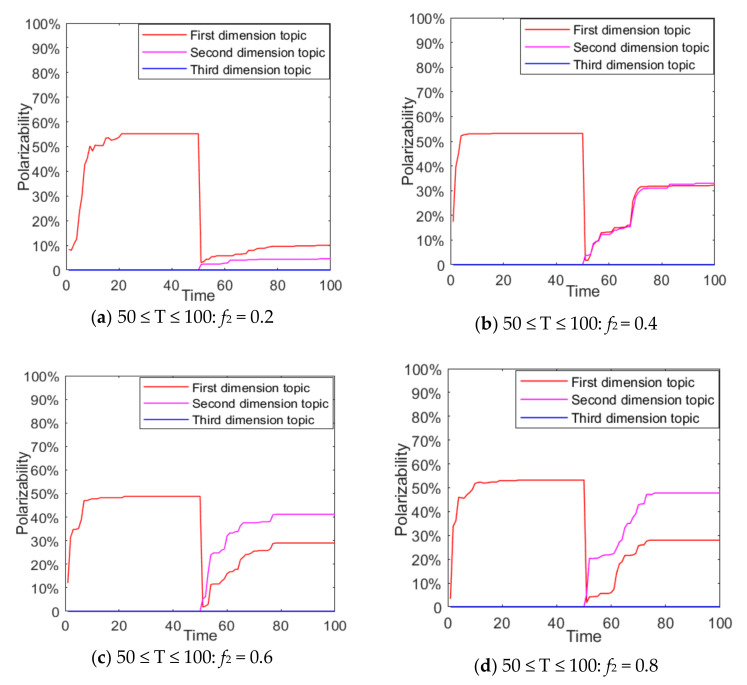
The polarizability curve of group attitude under different external information intensity at different time.

The intervention of external information in different dimensions at different times usually promotes the derivation of public opinion topics. Analyzing the influence of external intervention information in the process of the derivation of public opinion topics is of great significance for the research of multi-dimensional public opinion polarization. First of all, in the initial state, it is assumed that there is only one hot topic transmission in the public opinion field, and external intervention information of different dimensions is released at different times to observe the derivation of public opinion topics. For the convenience of research, the topic in the initial state is called the original topic, and the topic emerging with external intervention information is called the derived topic. The results are shown in Figure 16 and Figure 17.

It can be seen from Figure 16 and Figure 17 that when T= 0 ~ 50 (T = time), there is only the dissemination of the first-dimensional topic information in the public opinion field. At this time, individuals in the group carry out discussions around the first-dimensional topic and an obvious polarization is formed. When T = 50 ~ 100, with the intervention of external positive information on topic 2, more individuals begin to participate in the discussion of the second dimension, forming a multidimensional public opinion polarization. Moreover, with the enhancement of the external positive information intensity of topic 2, the polarizability of topic 1 decreased, while that of topic 2 increased significantly. This shows that the intervention of external information in other dimensions can promote the derivation of topics, and with the enhancement of the information intensity of external intervention in the derivation topics, the polarizability of the original topics decreases continuously.

#### 4.4.2. The Influence of External Intervention Information on the Multi-Dimensional Multi-Stage Public Opinion Polarization Process

**Figure 18:**
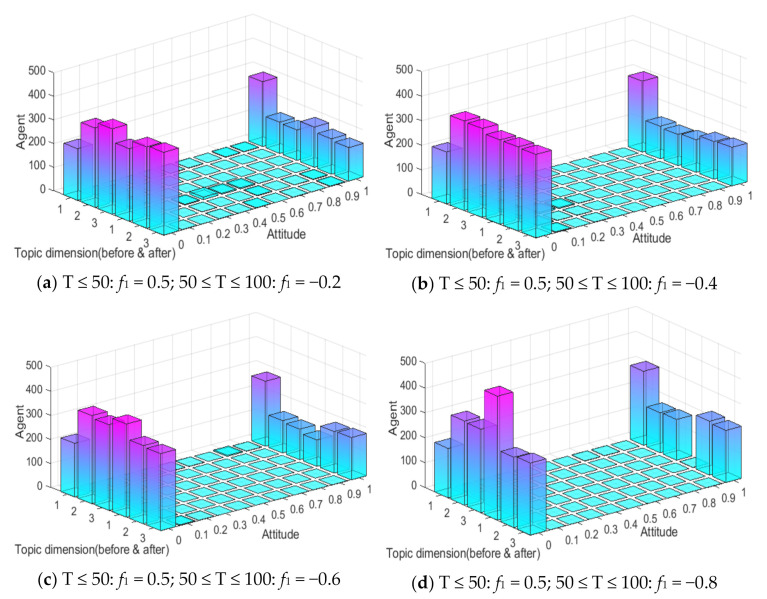
Histogram of group attitude distribution at different moments under different information intensity.

**Figure 19:**
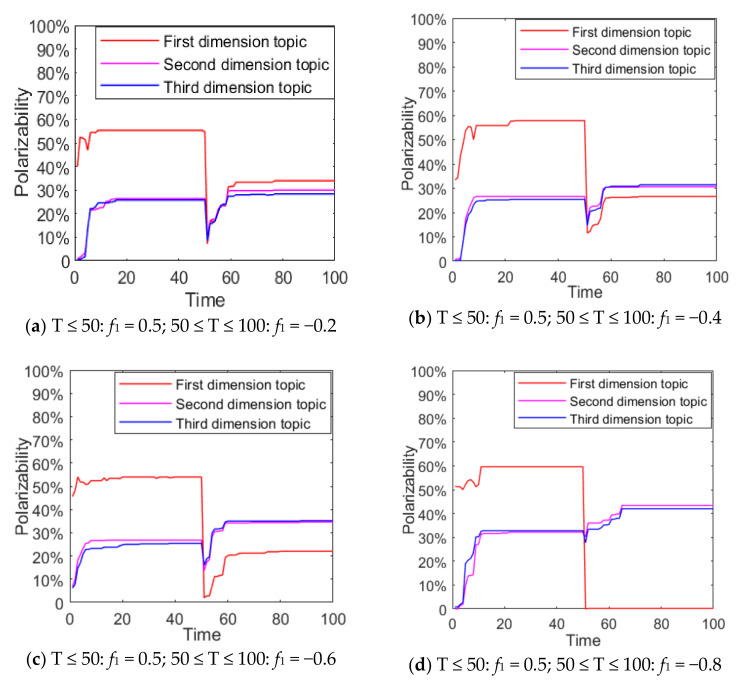
The polarizability curve of group attitude at different time under different information intensity.

In real life, due to the “authenticity” of network information, with the release of authoritative information the polarization process of network public opinion tends to take on a multi-stage form. The following is a study of multi-dimensional and multi-stage public opinion polarization phenomenon, and the results are shown in Figure 18 and Figure 19.

It can be seen from Figure 18 and Figure 19 that, when 0 ≤ T ≤ 50, since the external information intensity of the first dimensional topic is 0.5, the attitude polarizability of topic 1 is about 60%, and that of topic 2 and 3 is about 30%. When 50 ≤ T ≤ 100, with the intervention of external negative information, the group attitude of topic 1 moves towards value 0, and attitude polarizability gradually declines. However, topic 2 and 3 are affected positively, and their attitude polarizability are increased. The obvious public opinion inversion occurs during 0 ≤ T ≤ 50 and 50 ≤ T ≤ 100. This shows that when two completely opposite kinds of information are released by a certain dimensional topic at different moments, network public opinion will form a multi-dimensional and multi-stage polarization phenomenon, which is different from the public opinion inversion on a single dimensional topic. Multi-dimensional and multi-stage polarization phenomena can be regarded as individual conversion between multi-dimensional topics under the influence of external information.

**Figure 20:**
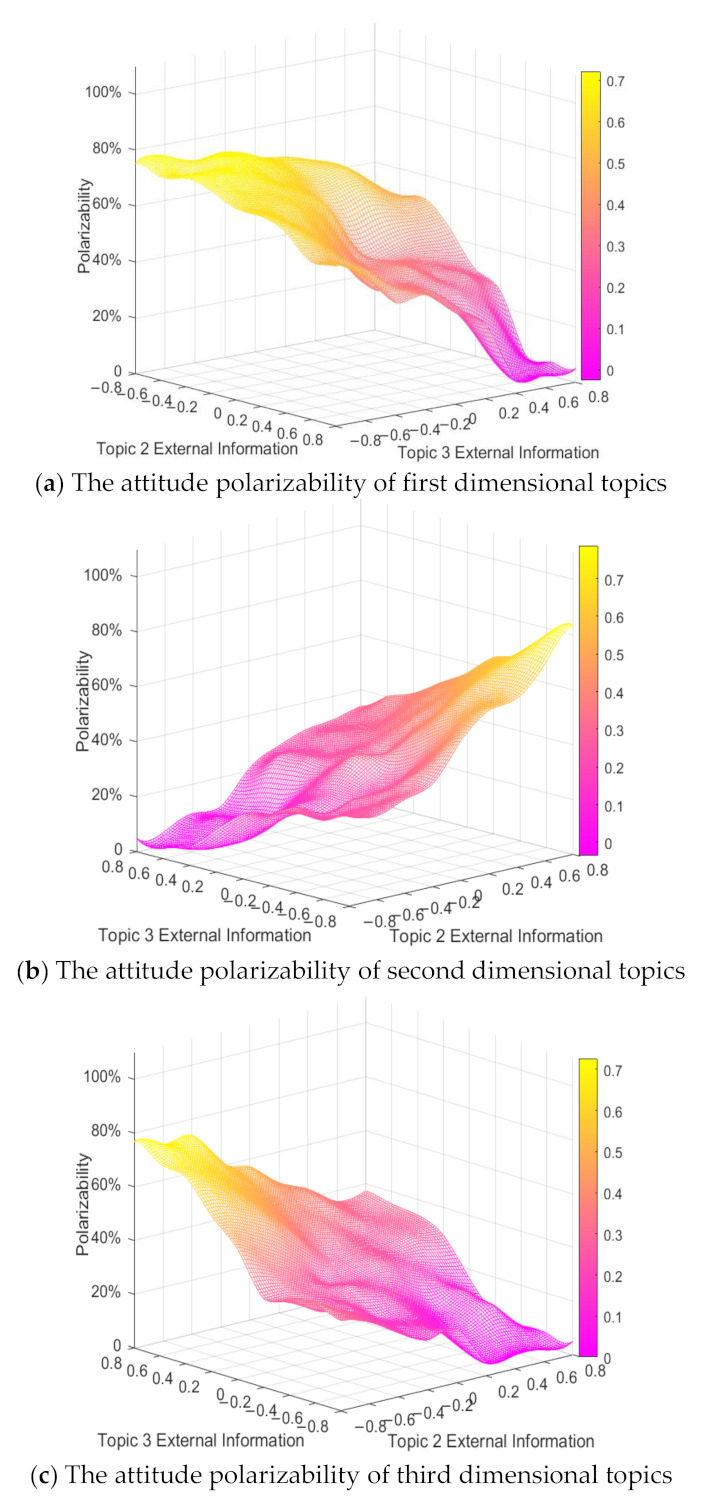
The attitude polarizability of external information intervention in topic 2 and topic 3 at different moments.

In addition, the multi-dimensional polarization process of external information intervention in any single dimension is discussed above. In the evolution process of public opinion, there are usually multiple dimensions of external information intervention, which will have more complex influences on the process. The external information intensity *f*_1_ = 0.5 of the first dimensional topic at time between 0 and 50, and *f*_2_ and *f*_3_ at time between 50 and 100 are set as −0.8, −0.6, −0.4, −0.2, 0, 0.2, 0.4, 0.6 and 0.8, respectively, so as to discuss the changes of multi-dimensional public opinion polarization process when the external information of topic 2 and topic 3 at time between 50 and 100 is involved simultaneously. The setting of other parameters is the same as above, and the result is shown in Figure 20.

As can be seen from Figure 20a, with the gradual enhancement of the external intervention information intensity of topic 2 and topic 3, the attitude polarizability of topic 1 decreased significantly, and when the external information intensity of topic 2 and 3 was greater than 0.4, the attitude polarizability of topic 1 decreased sharply to 0. This shows that when there is a negative correlation between multi-dimensional topics, the intervention of positive information about related topics will make the attitude polarizability of other dimensional topics fall sharply. It can be seen from Figure 20b that when the external information of topic 2 and topic 3 is released at the same time, the polarizability of topic 3 greatly increases with the enhancement of the external information intensity of topic 2. It can also be seen from Figure 20c that with the enhancement of the external information intensity of topic 3, the attitude polarizability of topic 3 also increases. Based on Figure 20a–c, it can be seen that with the enhancement of the external information intensity of *f*_2_ and *f*_3_, the attitude polarizability of topic 1 decreased sharply, while that of topic 2 and 3 increased accordingly. This indicates that the intervention of external information in different dimensions at different times will further form the multi-dimensional and multi-stage polarization of public opinion, and the more relevant topics and information intensity released by the information means that extent of public opinion inversion is greater. Therefore, in public opinion control, relevant departments should release authoritative information in a timely fashion about hot events, guide the public opinion to a certain correct dimension, and avoid the continuous impact caused by the event.

#### 4.4.3. The Combined Analysis of External Intervention Information and Topic Correlation

**Figure 21:**
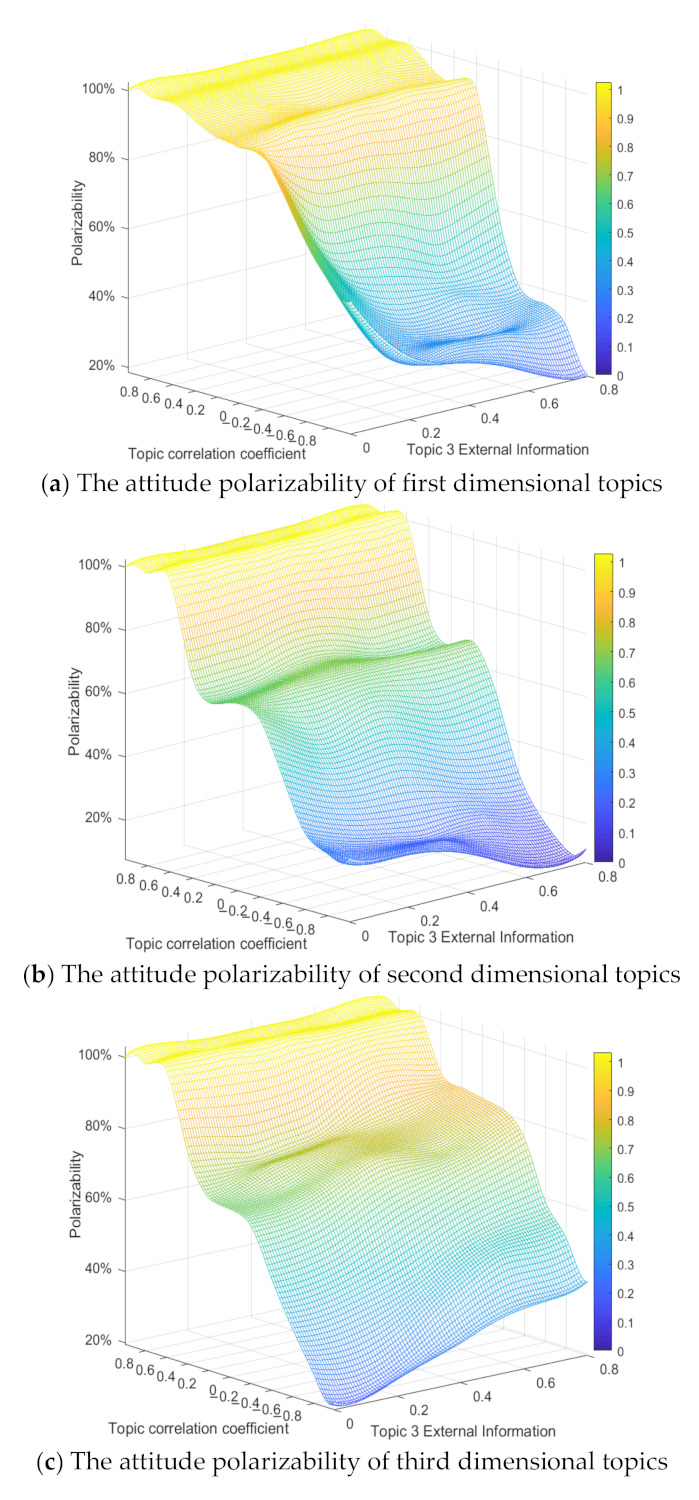
The relationship between external information intensity, topic correlation coefficient and attitude polarizability.

When there is a certain correlation between multi-dimensional topics, the external information intervention of any dimensional topic will have an impact on its related topics. Therefore, combined with the topic correlation coefficient, it is very important to analyze the influence of external intervention information on the multi-dimensional public opinion polarization process. Here, at the time moments between 0 and 50, the external information intensity *f*_1_ is set to 0.5. At the time moments between 50 and 100, the external information intensity *f*_3_ is set to 0.2, 0.4, 0.6 and 0.8, respectively, to observe the change of the polarizability of topic attitude in each dimension. In order to simplify the simulation process, only the situation when external positive information involved is analyzed here, and the conclusion can be extended to other cases. The results are shown in Figure 21.

In Figure 21, *x*-axis is the external information intensity of topic 3, *y*-axis is the topic correlation coefficient, and *z*-axis is the polarizability. With the decrease of the topic correlation coefficient, the attitude polarizability of topics in all dimensions decreases gradually. When the topic correlation coefficient is greater than 0, as its value increases, the attitude polarizability of topics in all dimensions keeps rising and tends to be stable. At this time, with the enhancement of the external positive information intensity of topic 3, the attitude polarizability of topic 1 and topic 2 does not change significantly, while that of topic 3 increases significantly. This shows that when there is a positive correlation between multi-dimensional topics, the intervention of external information on any dimensional topic will only have an impact on itself, with relatively small impact on its related topics. However, when the topic correlation coefficient is less than 0, with the enhancement of the external positive information intensity of topic 3, the polarizability of topic 1 and 2 decreases continuously, while the polarizability of topic 3 increases significantly. Therefore, when there is a negative correlation between multi-dimensional topics, the intervention of positive information outside the related topics will have a positive impact on itself and a negative impact on the related topics. On the whole, the topic correlation coefficient has a stronger influence on the multi-dimensional public opinion polarization process, and the influence of external intervention information on other dimensional topics will be constrained by the topic correlation coefficient.

## 5. Empirical Analysis

### 5.1. Selection of Empirical Cases and Data Acquisition

This paper selects a typical case “Tencent sues Laoganma” to verify the multi-dimensional public opinion polarization model constructed in this paper.

**Figure 22:**
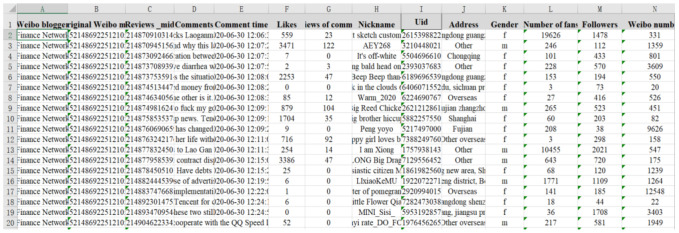
Weibo comments.

On 30 June 2020, Tencent requested the court to seize 16.24 million Yuan as “property preservation” due to an unpaid advertising fee. The news aroused strong attention among netizens. Laoganma responded that it “has no business cooperation with Tencent and called the police”. On 1 July, Guizhou police issued a notice saying that “someone pretended to be Laoganma and signed a cooperation agreement with Tencent, resulting in Tencent’s prosecution.” Relevant information caused hot discussion in multiple dimensions, such as: “Laoganma is faithless, defaulting huge advertising fee”, “Tencent were cheated”, “Nanshan District court freezes tens of millions”. Multi-dimensional discussion was carried out which can be summarized under the following three aspects: (1) relevant evaluation of Tencent; (2) relevant evaluation of The Godmother; (3) relevant evaluation of Nanshan District Court in Shenzhen. Based on this, this event is selected as an empirical case in this paper. Python crawler technology was used to obtain Weibo information published in “Toutiao”, “The Paper”, “CCTV news” and other media released between 30 June and 3 July 2020. There are total of 50,000 Weibo comments, including the publisher’s ID, posting time, number of followers, number of comments, number of likes, etc. The results are shown in Figure 22.

### 5.2. Data Processing

Since the acquired raw data contains a large amount of useless information, and the Chinese text data contains various error encoding information, the raw data needs to be processed. The specific steps are as follows:

Step1: Data cleaning. Python data cleaning tools are used to eliminate raw data containing large amounts of incorrect garbage, emoticons, missing or duplicate redundant information. In total, there are more than 30,000 useful pieces of information left.

**Figure 23:**
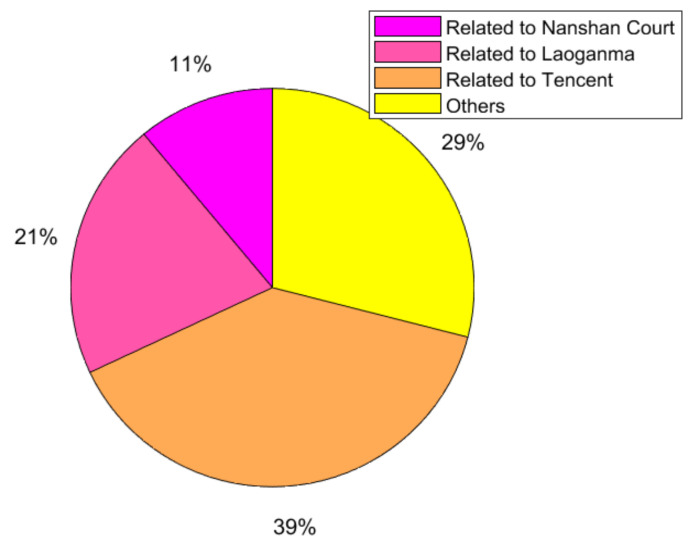
Classification of Weibo comments.

Step2: Text classification. By observing the data obtained, it can be seen that netizens’ comments on the event are roughly divided into three dimensions. However, because the dataset is large and messy, it needs to be classified as text. By comparing the three text classification methods of Recurrent Neural Network (RNN) [38], FastText (Facebook open source a simple and efficient text classifier) [39] and Convolutional Neural Network (CNN) [40], CNN has an accuracy of 82.41%, which is significantly better than the other two methods. Therefore, in this paper, CNN was finally selected for text classification of Weibo comment data into four categories:(1) related to Tencent; (2) related to Laoganma; (3) related to Nanshan District Court; (4) others; The specific distribution of the number of comments in the four categories is shown in Figure 23.

**Figure 24:**
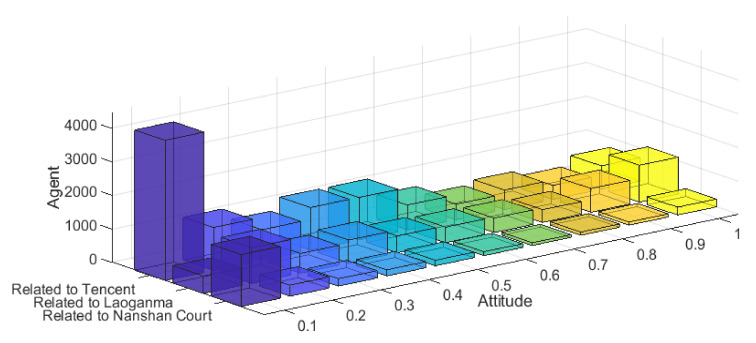
The distribution chart of public opinion.

Step3: Text Sentiment Scoring. The raw data crawled is the comment information in Chinese texts, and in order to correspond to the model, the Chinese texts need to be processed for sentiment scoring to quantify the sentiment tendency of individuals. Based on Step2, the “other irrelevant comments” category is excluded here, and more than 20,000 comments are selected. The sentiment score was processed by the natural language processing tool SnowNLP [41,42], the sentiment value of each Weibo comment was obtained by quantification, and the results are shown in Figure 24.

### 5.3. Analysis of Results

30,000 comments selected by convolutional neural network are divided into four categories, among which the comments “related to Tencent” account for 39% of the total, indicating that under the influence of related information, netizens have made many comments on Tencent’s behavior. Second, “Related to Laoganma” accounts for 21% of the comments, indicating that netizens have also paid great attention to “Laoganma”. At the same time, Shenzhen Nanshan District Court played an important role in the process of freezing Laoganma’s property, and due to the contradiction of relevant information before and after, many netizens questioned the behavior of Nanshan District Court and made many comments on it. Finally, other comments that had “nothing to do with the incident” accounted for 29%. In general, netizens usually comment on an event from multiple dimensions, which indirectly explains the actual value of the multi-dimensional public opinion polarization model constructed in this paper.

On this basis, this paper eliminated the category of “other irrelated comments” and selected a total of more than 20,000 comment data. Through SnowNLP, a natural language processing tool, emotion scoring was conducted to obtain the emotion value of each Weibo comment by quantification. The results are shown in Figure 24.

The evolution process of this event is dominated by three pieces of external information. First, “Tencent asked the court to seize Laoganma’s tens of millions on the grounds that it owed a huge amount of advertising fee”. This news immediately attracted the attention of some netizens and gave Tencent “support and sympathy”. Secondly, as the news broke that Laoganma had “no cooperation with Tencent”, netizens paid more attention to this information and began to show “sympathy” to Laoganma, while condemning Tencent’s behavior. Finally, with the announcement of the Guizhou police and the release of the “Tencent was cheated” information, many netizens began to question the Nanshan District Court’s freezing of Laoganma’s property. As can be seen from Figure 24, under the influence of the three parties’ information, netizens made comments on the event from different dimensions. Most netizens thought that “Tencent staff did not make detailed investigation prosecution” and made negative comments, while only a minority of Internet users expressed sympathy and support. However, most netizens thought that “Laoganma was bullied” and showed “sympathy and support”. At the same time, some netizens “questioned and disagreed with Nanshan District Court”. On the whole, under the influence of multiple information, netizens commented on the event from multiple dimensions and formed a multi-dimensional polarization of public opinion.

**Figure 25:**
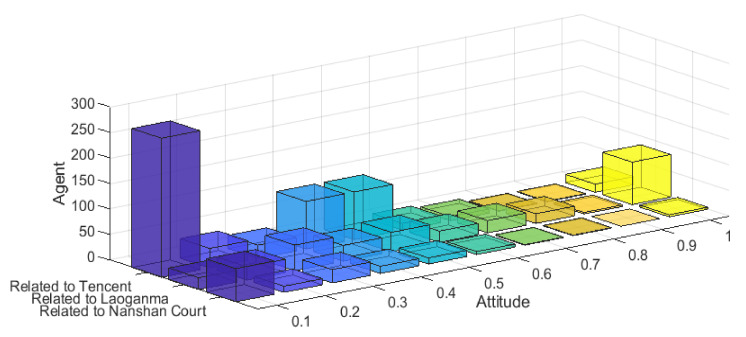
The distribution chart of public opinion based on constructed model.

The following is the simulation of the event according to the multi-dimensional public opinion polarization model proposed in this paper. Due to the large amount of data and the comprehensive consideration of visualization, this paper sets the network size of the simulation as 1000, and sets the initial group size in the simulation according to the proportion of the three types of comments. Since the event was dominated by three external pieces of intervention information, *f*_1_ = 0.2 (indicating the intensity of external information related to Tencent) was set in the simulation. *f*_2_ = 0.5 (indicating the intensity of external information related to Laoganma); *f*_3_ = 0.1 (represents external information relevant to Nanshan District Court). At the same time, the correlation of topics in three dimensions is set as follows: the topic of “Tencent” and “Laoganma” shows strongly negative correlation; the topic of “Laoganma” and “Nanshan District Court” shows strongly negative correlation; the topic of “Tencent” and “Nanshan District Court” shows moderately positive correlation. Other parameters are set as follows: the individual point confidence C is subject to N ~ (0.5, 0.2) and mapped to [0, 1], indicating that most individuals’ point confidence is general. *d*_1_ = 0.15, *d*_2_ = 1.15, *μ* = 0.35, *β* = 0.15; Interaction time T = 20; the result is shown in Figure 25.

It can be seen from Figure 25 that the model proposed in this paper simulates the public opinion distribution of the event appropriately. First of all, for topics with “Tencent-related” dimension, the model not only simulates the attitude distribution of netizens towards this topic on the whole, but also reasonably describes the extreme individuals distributed in the case. For the topic “Laoganma-related”, the model simulates its overall attitude distribution, which is basically consistent with the netizens’ attitude distribution in the case. As for the dimensional topic “Nanshan District Court-related”, most netizens’ comments on this topic are relatively “negative”, which is also consistent with the attitude distribution in the case. On the whole, the multi-dimensional public opinion polarization model can simulate well public opinion events in reality, which has certain practical significance for analyzing the causes of multi-dimensional public opinions and predicting the evolution trend of public opinions.

**Figure 26:**
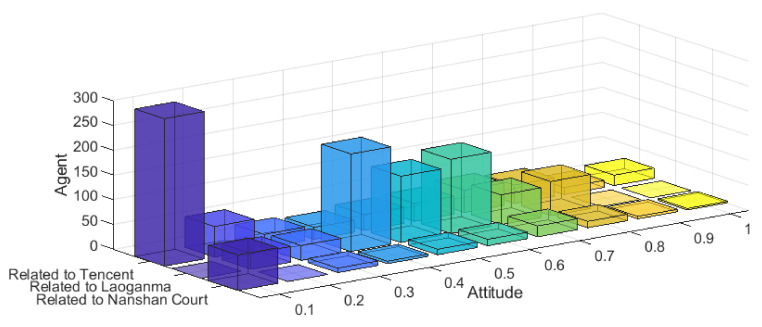
The distribution chart of public opinion proposed by the literature [25].

In order to further compare the differences with other models, a model in the literature [25] was used to simulate the “Tencent Sues Laoganma”, and the results are shown in Figure 26.

Although the literature [25] described distribution of netizen’s attitudes, the description of extreme individuals on different dimensional topics is not accurate. The simulated and actual data on topics “related to Laoganma” is quite different, which does not reflect the distribution of the extreme individuals. Therefore, through comparison, it can be seen that the model proposed in this paper is more in line with the multi-dimensional public opinion evolution in reality.

## 6. Conclusions

In order to reveal the evolution mechanism of the polarization of multidimensional public opinion based on a topic derivative situation, this article considers the interaction mechanism between different dimensions of subject and multidimensional interaction rules, and builds a multidimensional public opinion polarization model so as to analyze the influence of individual participation status, individual opinion confidence, topic correlation coefficient, and external intervention information on the polarization process of multidimensional public opinion.

The following conclusions can be drawn from the simulation experiments:

1. When there is a negative correlation among multi-dimensional topics, as the opinion of participants from different dimensions is gradually the same, the conflict between multi-dimensional topics will weaken the overall polarization effect of public opinion, while when there is a positive correlation between multi-dimensional topics, the polarization effect of public opinion will be correspondingly enhanced.
2. When there is a negative correlation between multi-dimensional topics, with the increase of opinion confidence of an individual, the interaction between topics of different dimensions will weaken accordingly, thus enhancing the effect of public opinion polarization.
3. When multi-dimensional topics are positively correlated, the participation of a certain proportion of opinion leaders can promote more individuals to participate in the discussion of multi-dimensional topics. However, when multi-dimensional topics are negatively correlated, the interaction between different dimensional topics will cause individuals to give up their support for a certain dimensional topic, thus reducing their participation in multi-dimensional topics.
4. The intervention of external intervention information in different dimensions at different times will further form the multi-dimensional and multi-stage public opinion polarization phenomenon; however, the influence of external intervention information on multi-dimensional topics is constrained by the topic correlation coefficient, i.e., when multi-dimensional topics are negatively correlated, the intervention of external intervention information has a stronger influence on the multi-dimensional public opinion polarization process.

However, there are still some deficiencies in this paper, which need further study:

1. Although this paper considers the interaction between topics in different dimensions and defines the topic correlation coefficient to describe it, it does not give a specific calculation method, so it is necessary to artificially determine the correlation coefficient between topics in different dimensions. Therefore, in follow-up research, big data and AI technology can be used to comprehensively analyze various factors among topics of different dimensions and calculate the correlation coefficient between topics.
2. As the spread of social hot events is usually a dynamic process, individuals participating in the discussion of events in the network will enter and exit, so it is necessary to consider the increase and withdrawal mechanism of nodes in the network and study the phenomenon of multi-dimensional public opinion polarization in the dynamic network.

## Figures and Tables

**Table 1:** Relevant parameters.

$C\left(i\right)$	opinion confidence of individual *i*
*d*_1_	assimilation effect zone distance
*d*_2_	repulsion effect zone distance
*μ*	assimilation parameter
*β*	repulsion parameter
*ρ_mn_*	relevant parameter between the mth dimensional topic and the nth dimensional topic
$f_{m}$	external information intensity of the mth dimensional topic

**Table 2:** Relevant parameters.

$x_{n}\left(i\right)$	the attitude value of individual i to the nth dimensional topic
${\dot{x}}_{n}\left(i\right)$	attitude value presented by individual i after the change in support for the nth dimensional topic
${\ddot{x}}_{n}\left(i\right)$	attitude value of individual i after the interaction with individual *j*
$S_{n}\left(i\right)$	Individual’s support degree of the nth dimensional topic
$e_{n}^{m}$	the impact of the mth dimensional topic on the nth dimensional topic
$E_{n}^{m}$	the intensity of the impact of the mth dimensional topic on the nth dimensional topic
$F_{n}^{m}$	the intensity of the impact of external intervention information of the mth dimensional topic on the nth dimensional topic

**Table 3:** The topic correlation coefficient *ρ_mn_* between topics of different dimensions.

Topic Correlation Coefficient *ρ_mn_*	The Degree to Which the *m*th Topic Is Related to the *n*th Topic
*ρ_mn_* ∈ [−1, −0.8)	Extremely strong negative correlation
*ρ_mn_* ∈ [−0.8, −0.6)	Strong negative correlation
*ρ_mn_* ∈ [−0.6, −0.4)	Medium negative correlation
*ρ_mn_* ∈ [−0.4, −0.2)	Weak negative correlation
*ρ_mn_* ∈ [−0.2, 0)	Extremely weak negative correlation
*ρ_mn_* = 0	Independent
*ρ_mn_* ∈ (0,0.2]	Extremely weak positive correlation
*ρ_mn_* ∈ (0.2,0.4]	Weak positive correlation
*ρ_mn_* ∈ (0.4,0.6]	Medium positive correlation
*ρ_mn_* ∈ (0.6,0.8]	Strong positive correlation
*ρ_mn_* ∈ (0.8,1]	Extremely strong positive correlation

## Data Availability

The data used to support the findings of this study are available from the corresponding author upon request.
